# Diet-driven mercury contamination is associated with polar bear gut microbiota

**DOI:** 10.1038/s41598-021-02657-6

**Published:** 2021-12-03

**Authors:** Sophie E. Watson, Melissa A. McKinney, Massimo Pindo, Matthew J. Bull, Todd C. Atwood, Heidi C. Hauffe, Sarah E. Perkins

**Affiliations:** 1 School of Biosciences, Cardiff University, The Sir Martin Evans Building, Museum Avenue, Cardiff, UK; 2 Research and Innovation Centre, Fondazione Edmund Mach (FEM), Via E. Mach 1, 38098 San Michele all’Adige, Italy; 3 Department of Natural Resource Sciences, McGill University, Ste-Anne-de-Bellevue, QC Canada; 4 U.S. Geological Survey (USGS), University Drive, Anchorage, USA

**Keywords:** Ecology, Conservation biology, Microbial ecology, Molecular ecology

## Abstract

The gut microbiota may modulate the disposition and toxicity of environmental contaminants within a host but, conversely, contaminants may also impact gut bacteria. Such contaminant-gut microbial connections, which could lead to alteration of host health, remain poorly known and are rarely studied in free-ranging wildlife. The polar bear (*Ursus maritimus*) is a long-lived, wide-ranging apex predator that feeds on a variety of high trophic position seal and cetacean species and, as such, is exposed to among the highest levels of biomagnifying contaminants of all Arctic species. Here, we investigate associations between mercury (THg; a key Arctic contaminant), diet, and the diversity and composition of the gut microbiota of polar bears inhabiting the southern Beaufort Sea, while accounting for host sex, age class and body condition. Bacterial diversity was negatively associated with seal consumption and mercury, a pattern seen for both Shannon and Inverse Simpson alpha diversity indices (adjusted R^2^ = 0.35, F_1,18_ = 8.00, P = 0.013 and adjusted R^2^ = 0.26, F_1,18_ = 6.04, P = 0.027, respectively). No association was found with sex, age class or body condition of polar bears. Bacteria known to either be involved in THg methylation or considered to be highly contaminant resistant, including Lactobacillales, Bacillales and Aeromonadales, were significantly more abundant in individuals that had higher THg concentrations. Conversely, individuals with higher THg concentrations showed a significantly lower abundance of Bacteroidales, a bacterial order that typically plays an important role in supporting host immune function by stimulating intraepithelial lymphocytes within the epithelial barrier. These associations between diet-acquired mercury and microbiota illustrate a potentially overlooked outcome of mercury accumulation in polar bears.

## Introduction

The gut microbiota (the complex community of microorganisms within the gut) plays a critical role in the regulation of physiological functions, and possibly also in metabolism and toxicity of environmental contaminants, in the host^1^. In particular, the microbiota is likely an important mediator for toxicity of heavy metals (see Guo et al.^2^ and Dempsey et al.^3^), with evidence of bacterial demethylation in the gut contributing to the elimination of mercury from a host^4,5^. In humans, *Sutterella parvirubra* and *Acidaminococcus intestini* within the gut have been shown to degrade compounds containing mercury^2^. Additionally, contaminants may negatively impact gut bacteria via effects on diversity and/or composition of bacterial communities. In vivo studies demonstrate that in laboratory mice and rats, a suppressed or depleted gut microbiota community is associated with lower faecal excretion of mercury and increased accumulation of mercury in host tissues^6–8^. In the isopod *Porcellio scaber,* for example, individuals from mercury-polluted compared to unpolluted environments demonstrated a lower bacterial species richness in the gut, as well as elevated levels of bacteria from the genera *Pseudomonas*, *Listeria* and the phylum Bacteroidetes^9^. In laboratory mice exposed to high mercury levels, the abundance of *Sporosarcina* sp., *Jeotgailcoccus* sp., and *Staphylococcus* sp. were significantly decreased in treatment groups relative to controls^10^. It is therefore possible that contaminant-induced alterations to the composition of gut bacteria may influence the toxicity of contaminants and, ultimately, impact host health^10^. This, alongside other well-documented physiological effects of contaminants^11,12^ makes gut microbiota-contaminant interactions relevant for species survival, especially for top predators that can bioaccumulate high levels of contaminants from their ingested prey.

The polar bear (*Ursus maritimus*) is a long-lived, wide-ranging apex predator, which feeds on a variety of high trophic position seal and cetacean species^13–15^. As such, polar bears through their diet are exposed to among the highest levels of biomagnifying contaminants^16^, including methylmercury (MeHg), of all the Arctic species. After climate-driven loss of sea ice habitat, contaminant exposure is considered to be one of the most significant threats to polar bears^16^, owing to the severe detrimental effects it has on host health^12,16,17^. In the past century, concentrations of mercury have increased substantially in the Arctic, with 94% of the mercury found in polar bear tissues estimated to be derived from anthropogenic sources, resulting from long-range transport to the Arctic^18^. Since contaminants transported to the Arctic persist within the food chain^19,20^, any impact of MeHg in polar bears is a sign of a much wider problem in the Artic at large.

In itself, diet is considered an important determinant of changes to the gut microbiota^21^. Gut microbiota analysis of humans and 59 other mammalian species indicates that host diet has a strong influence on bacterial diversity and composition, which increases from carnivory to omnivory to herbivory^21^. In free-roaming brown bears (*Ursus arctos*), gut microbiota composition changes seasonally, which is thought to reflect extreme seasonal shifts in dietary intake^22^. Similarly, in Andean bears (*Tremarctos ornatus*), gut microbiota richness differs between captive and wild types, which is thought to reflect differences in the availability and diversity of food resources^23^. For polar bears, substantial changes in the spatial and temporal extent of sea ice habitat since *c*. 2000 have led to shifts in prey availability and recruitment^24,25^. Polar bears primarily consume a diet of seals, namely ringed seals (*Pusa hispida*) and bearded seals (*Erignathus barbatus*)^14,15^, which occupy high trophic positions^26^ and can accumulate high levels of contaminants^27–29^. Such diet-driven exposure to contaminants, including mercury^30^, may in turn may have a knock-on effect on gut microbiota diversity and composition. Previous work demonstrated that climate-driven changes in habitat use by polar bears is associated with differences in their gut microbial communities, suggesting that these differences may reflect the differing diets and contaminant exposures between habitats^31^. However, the potential link between diet-driven contaminant exposure and variation in the gut microbial communities of polar bears remains unstudied. Ringed and bearded seals are considered the “traditional” prey of polar bears, and so poorer access to these prey items due to reductions in sea ice extent and availability may have implications for polar bear gut microbial communities. Here, we investigate associations between tissue mercury concentrations, diet, and the faecal microbiota diversity and composition of Southern Beaufort Sea polar bears.

## Methods

### Polar bear capture and sampling

Polar bears (*Ursus maritimus*) were captured under the United States Geological Survey (USGS) Polar Bear Research Program (Marine Mammal Permit MA690038 to T.C.A.) in an area ranging approximately from Utqiağvik, Alaska (156° W) in the west to Demarcation Point (140° W) at the US-Canada border in the east (see Watson et al. 2019^31^ for sampling methods). Capture protocols were approved by the U.S. Geological Survey Institutional Animal Care and Use Committee. All methods were performed in accordance with relevant guidelines and regulations. Faecal samples from a total of 91 bears were obtained in years 2008, 2009, 2010 and 2013 and analysed previously for gut microbiota^31^. A single faecal sample was collected directly from the rectum of polar bears using a sterile latex glove and immediately transferred to a sterile Whirlpak bag (Nasco, Fort Atkinson, Wisconsin, USA) for storage. All faecal samples were stored at − 20 °C for the duration of the field season (up to ~ 5 weeks) before being stored at − 80 °C at the USGS, Alaska Science Center (Anchorage, Alaska, USA), and subsequently shipped on dry ice to the Fondazione Edmund Mach, Italy (CITES permit IT/IM/2015/MCE/01862 to S.W.) for metataxonomic analysis. Age of subadults and adults was estimated by extracting and analysing the cementum annuli of a vestigial premolar tooth^32^ and body condition for each polar bear was estimated using a ‘Body Condition Index’ metric^33^.

Due to opportunistic sampling, not all individuals had both hair and adipose tissue samples collected. Hair samples for mercury analysis were available for 63 of the individuals (2008–2010 samples previously reported in McKinney et al.^30^; some of the 2013 samples, previously reported in Bourque et al.^15^). Adipose tissue samples for fatty acids-based analysis of diet were available for 50 individuals (2008–2010 samples reported in McKinney et al.^14^; 2013 samples reported in Bourque et al.^15^). An adipose tissue sample was taken from the rump of 50 individual polar bears using a 6 mm biopsy punch. All adipose tissue samples were stored at − 80 °C prior to shipment to the McKinney lab for diet analysis. For 22 individuals, both hair for mercury analysis and adipose tissue for diet analysis were available (see Supplementary Table 1 for a breakdown of the samples and details of samples regime across individuals).

### Extraction of bacterial DNA and 16S rRNA gene amplification and sequencing

Full methods used for extraction and amplification of bacterial DNA was detailed in Watson et al.^31^. In brief, all faecal matter was collected from inside each sample glove using a sterile cotton swab (APTACA sterile transport swabs, Brescia, Italy). The swab was subsequently vortexed in phosphate-buffered saline solution (PBS) and pelleted by centrifugation. Lysis buffer and stainless steel beads (Qiagen) were added to each sample before homogenisation, followed by shaking at 37 °C for 40 min. Microbial DNA was extracted using the QIAamp^®^ DNA Mini Kits (QIAGEN©, Milan, Italy), following the manufacturer’s Buccal Swab Spin Protocol for cotton swabs (QIAamp^®^ DNA Mini and Blood Mini Handbook), but starting from step 2 (addition of Proteinase K). Extraction controls were included to control for contamination. The primer set 341F (5′-CCTACGGGNGGCWGCAG-3′) and 805Rmod (5′-GACTACNVGGGTWTCTAATCC-3′) (based on Klindworth et al.^34^ with degenerate bases) with overhanging Illumina adaptors, were used to target a ~ 460 base pair (bp) fragment of the 16S rRNA gene (variable region V3–V4). PCR products were purified and Illumina^®^ Nextera XT indices and sequencing adaptors (Illumina^®^) were incorporated using seven cycles of PCR (16S Metagenomic Sequencing Library Preparation, Illumina^®^). Final quantified libraries were pooled in equimolar concentrations before sequencing on an Illumina^®^ MiSeq (2 × 300 bp reads) at the Sequencing Platform, Fondazione Edmund Mach, Italy.

### Bioinformatic analyses

As detailed in Watson et al.^31^, reads were processed with MICCA v1.5.0^35^. Briefly, a total of 511,952 reads were detected across the samples. The data were rarefied to an equal depth within 90% of the minimum observed sample size (specifically 4571 reads per sample). Paired-end reads were merged, and pairs diverging by more than 8 bp or overlapping by less than 100 bp were discarded. PCR primers were trimmed and sequences not containing both PCR primer sequences were discarded. Finally, sequences were quality filtered at 0.5% Expected Error (EE); those displaying greater than 0.5% EE were discarded along with those shorter than 400 bp or containing unknown base calls (N). Using the VSEARCH cluster_smallmem algorithm^36^, operational taxonomic units (OTUs) were created de novo by clustering sequences with 97% sequence identity, discarding chimeric sequences. Taxonomic assignments of representative sequences from each OTU were performed using the RDP Classifier v2.12 in conjunction with RDP 16S rRNA training set 15^37^. OTU sequences were aligned and phylogenetic analysis was performed using Nearest Alignment Space Termination (NAST) and a phylogeny reconstructed using FastTree^38^, both via MICCA^35^. The raw sequencing data can be found at the National Centre for Biotechnology Information (NCBI) Sequence Read Archive (SRA) [Accession number: PRJNA542176].

### Mercury analysis

Total mercury (THg) concentrations were determined in 63 available hair samples, 39 of which were analysed and reported previously^30^. In brief, surface contamination was removed from each hair sample using standard protocols^18^ and then samples were acid digested, followed by THg analysis conducted by cold-vapour atomic absorption spectrometry. Blanks, sample duplicates, matrix spikes, and standard reference materials (DORM-3 and DOLT-4, National Research Council of Canada) were included as quality-control procedures, with all blanks demonstrating levels below the detection limit (i.e. less than 0.3 μg g^–1^, the limit based on an average sample size of ∼ 0.001 g). Precision was indicated by relative standard deviation of duplicate samples between 9 and 21%. Recoveries of matrix spikes ranged from 78 to 111%. Accuracy was indicated by THg concentrations measured in DORM-3 and DOLT-4 of 85 ± 16% and 93 ± 10% of the certified values, respectively. For the 24 samples not previously analysed and reported by McKinney et al.^30^, these were accurately weighed in a tared vessel and THg was quantified with a direct thermal composition mercury analyzer (NIC MA-3000, Nippon Instruments, Tokyo, Japan) (see Golzadeh et al.^39^). Quality control measures included at least one blank, a sample triplicate, and standard reference material (SRM: NIES 13) after every batch of 10 samples. Precision was indicated by relative standard deviation of triplicate samples between 6 and 28%. Accuracy was indicated by THg concentrations measured in NIES 13 of 4.60 ± 4.75% of the certified values. All blanks demonstrating levels below the detection limit (i.e. 0.3 μg g^–1^, the limit based on an average sample size of ∼ 0.001 g). Concentrations of THg were reported in μg g^–1^ dry weight (dw).

### Fatty acid-based diet analysis

The individual fatty acid composition of a given prey species (i.e. its fatty acid profile or signature) is incorporated into the tissues of the consumer (here a polar bear) largely unaltered. Based on this principle, by analysing the fatty acid composition of a polar bear’s adipose tissue, and comparing it to its potential prey, it is possible to make inferences about what prey species a given bear has consumed, as well as what proportions of that prey species has been consumed, referred to as Quantitative Fatty Acid Signature Analysis (QFASA)^40^. Here, QFASA was used to generate proportional diet estimates (% biomass) of bearded seal, beluga whale, bowhead whale and ringed seal for 50 individuals for which gut microbiota data was available. Fatty acid extraction and analysis for these samples was already reported (McKinney et al.^30^ and Bourque et al.^15^). In brief, the full adipose biopsy, minus the skin and hair, was accurately weighed and then extracted according to the Folch method. Extracted fatty acids were derivatised to fatty acid methyl esters (FAMEs) prior to analysis by gas chromatography with flame ionization detection (GC-FID). Each FAME was calculated as the mass% of total FAME.

### Statistical analyses

Data were analysed using R, version 3.5.2^41^. As QFASA generates proportional diet estimates (% biomass) of a given species within individuals, there is a strong degree of collinearity across each of the diet variables. Since low beluga and bowhead whale proportions were recorded within the polar bears studied here, only total seal consumption (i.e. combined proportions of bearded and ringed seal) was included in downstream modelling and analyses. This follows Bourque et al.^15^ who found that seal consumption remains the predominant prey type in Southern Beaufort Sea polar bears and is corroborated by a preliminary Principal Component Analysis (PCA) which we conducted to highlight the most important diet types explaining the variation in our data. PCA revealed that two principal component variables (PC1 and PC2) explained 78.77% (cumulative proportion = 0.7877) of the variation across the four dietary variables. A polar bear with a high PC1 value has a high proportion of bearded seal in their diet (PC1 = 0.6427) while a polar bear with a high PC2 value has a high proportion of ringed seal in their diet (PC2 = 0.7991).

Generalized linear models (GLMs) were used to investigate associations between Shannon and Inverse Simpson measures of bacterial alpha diversity and the sex, age class, body mass index, total mercury level, total proportion of seal consumption (i.e. combined proportion of bearded and ringed seal) and an interaction between mercury level and seal consumption. A gamma family and identity link function was used to model a Shannon measure of alpha diversity, while an inverse gaussian family and log link function were used to model an Inverse Simpson measure of alpha diversity. Three outlying data points were omitted from the model as their values fell more than three standard deviations from the mean. Model selection was based on fit of residual plots^42^ and backwards stepwise deletion of non-significant terms using AIC. No polar bears used within our analyses were repeat-sampled and therefore no multi-capture data is included.

The revised human hair no-observed-effects-level (NOEL) threshold for THg is 6.0 μg g^–1^ dw^43^ which we use as a threshold, above which polar bear mercury levels are deemed to have a detrimental impact on health^12,44^. The differential abundance of OTUs for polar bears above versus below the ‘safe threshold’ was examined using the R package ‘DESeq2’^45^. To illustrate potential effects of mercury microbiota composition we constructed a heat tree, using the log_2_ ratio of median proportions for bacterial taxa associated with polar bears with mercury levels below and above threshold levels using the R package, Metacoder^46^. The differential abundance of OTUs between polar bears with ‘high’ versus ‘low’ proportions of total dietary seal, were also examined using the R package ‘DESeq2’^45^. Dietary proportion was considered ‘high’ if the measurement detected was above or equal to the median across our samples, and considered ‘low’ if below the median. Data were analysed as categorical variables to adhere to DESeq2 package requirements.

### Animal handling

Polar bears were captured in accordance with relevant guidelines and regulations under the United States Geological Survey (USGS) Polar Bear Research Program (Marine Mammal Permit MA690038 to T.C.A.). Capture protocols were approved by the U.S. Geological Survey Institutional Animal Care and Use Committee. All methods were performed in accordance with relevant guidelines and regulations. The study is reported in accordance with ARRIVE guidelines.

## Results

### Associations between mercury, diet and gut microbiota diversity

**Figure 1:**
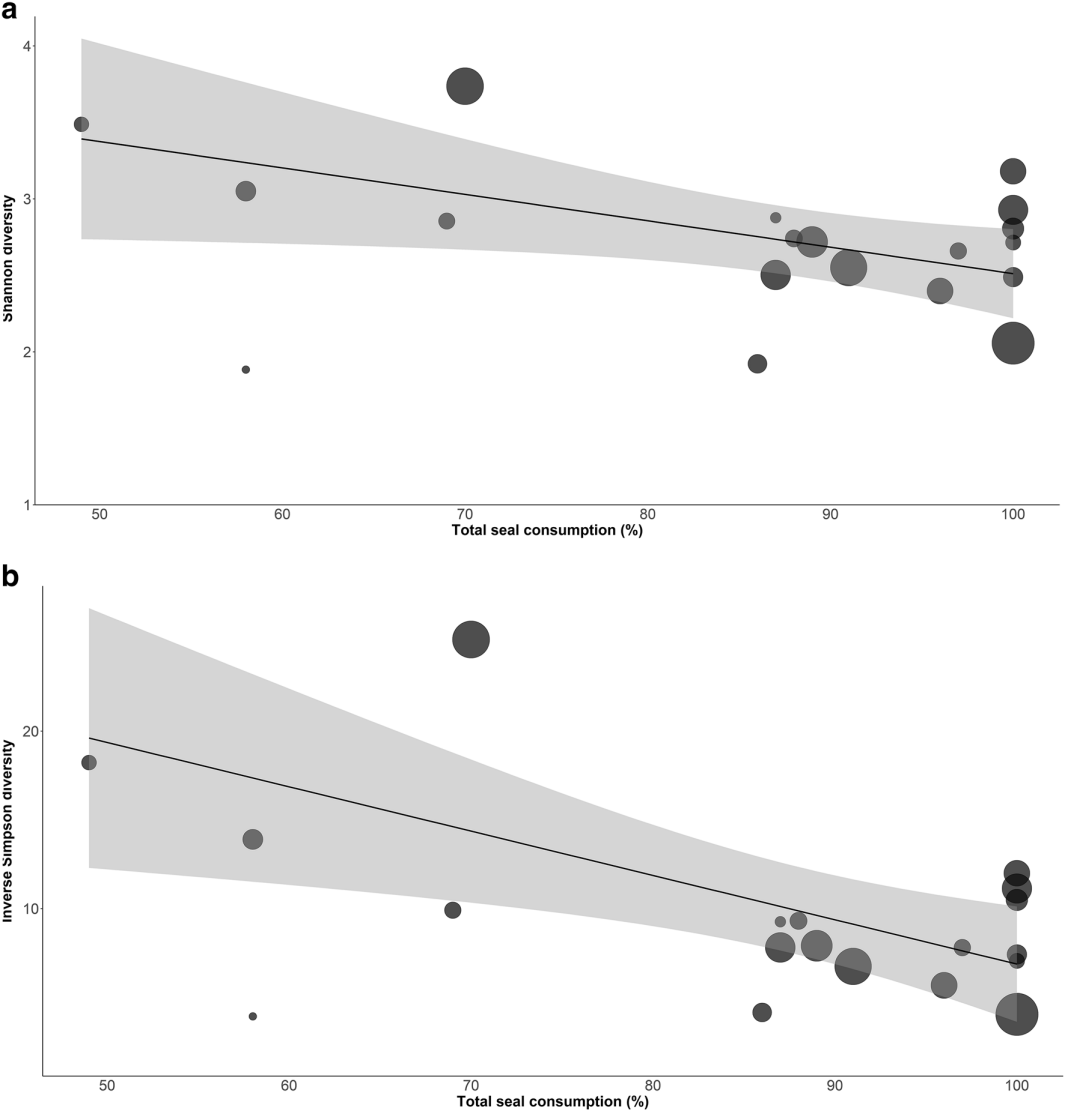
Associations between bacterial alpha diversity of the gut microbiota and percentage of seal consumed in the diet of Southern Beaufort Sea polar bears. Data points are weighted by hair total mercury concentrations measured for each individual, where a larger circle size reflects a higher mercury level detected within a given polar bear. The line of best fit takes in to account the weight of the data points.

Bacterial diversity was negatively associated with an interaction between proportions of seal consumed and hair THg concentrations; a pattern seen for both a Shannon and Inverse Simpson measure of alpha diversity (adjusted R^2^ = 0.35, F_1,18_ = 8.00, P = 0.013 and adjusted R^2^ = 0.26, F_1,18_ = 6.04, P = 0.027, respectively; Fig. 1a,b). No association was found between bacterial diversity and the sex, age class or body condition of polar bears (Supplementary Table 2).

### Associations between mercury and gut microbiota composition

Hair THg concentrations in individual polar bears ranged widely from 0.9 to 22.9 μg g^−1^ dw, with a mean of 4.7 μg g^−1^ dw, (SE = 0.45), which is below the ‘safe’ mercury threshold of 6.0 μg/g^43^. However, mercury levels ≥ 6.0 μg/g were detected in n = 16 of 63 individuals (25%). Differences in specific bacterial taxa were detected in individuals falling above versus below threshold levels for mercury, with 12 phyla detected in individuals above the threshold, compared to 20 phyla for those below [of which 9 phyla were unique for the latter (Acidobacteria, Candidate division WPS-2, Chlamydiae, Chloroflexi, Deferribacteres, Gemmatimonadetes, Lentisphaerae, Planctomycetes, Tenericutes)].

**Figure 2:**
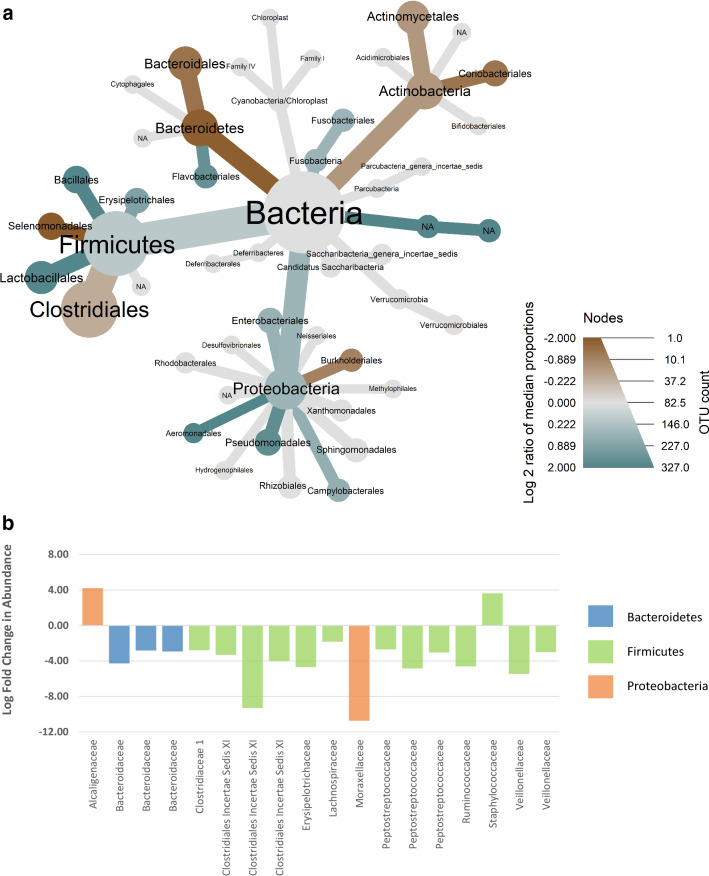
(**a**) Metacoder heat tree showing the log_2_ ratio of median proportions for bacterial taxa in polar bears demonstrating mercury concentrations above, compared to below, the 6.0 g g^–1^ dw no observed effect level (NOEL) threshold for humans. Nodes are weighted by OTU count and coloured on a gradient from largest decrease in median proportion (teal) to largest increase in median proportion (tan). (**b**) Differential OTU abundance from DESeq2 analysis of polar bears demonstrating mercury levels above, compared to below, NOEL threshold for humans. OTU are plotted at family level with associated phyla assignment.

For a number of taxa, the log fold ratios of median proportions per particular bacterial taxa differed between individuals above versus below the threshold for mercury (Fig. 2). Median proportions of Firmicutes and Proteobacteria were higher, and Bacteroidetes and Actinobacteria lower, in individuals above the threshold for mercury (Fig. 2a). At order level, the largest differences in median proportion were seen in Lactobacillales, Bacillales, Aeromonadales and an unclassified taxonomic class (which were higher in individuals above the threshold for mercury) and Bacteroidales, Selenomonadales and Coriobacteriales (which were higher in individuals below the threshold for mercury; Fig. 2a). DESeq analysis revealed that 18 OTUs differed significantly between bears above versus below the THg threshold. Specifically, the abundance of 16 OTUs was significantly lower in bears above the mercury threshold (the majority of which were assigned to families within the phylum Firmicutes), while the abundance of two OTUs were significantly higher in bears above the mercury threshold (Fig. 2b; Supplementary Table 3).

### Associations between diet and gut microbiota composition

**Figure 3:**
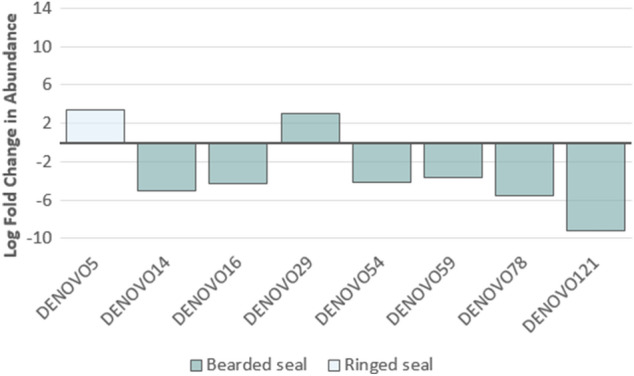
Log fold differential change in OTU abundance of polar bears demonstrating low levels of dietary bearded seal or ringed seal compared to those that demonstrated high levels. Dietary proportion was considered ‘high’ if the measurement detected was above or equal to the median across samples, and ‘low’ if below the median. Taxonomic information related to each OTU is given in Table 1.

DESeq analysis revealed that a number of OTUs significantly differed in their relative abundance depending on the proportion of seal species in an individual’s diet (Fig. 3; Table 1). Specifically, seven OTUs differed significantly between polar bears that had low proportions of bearded seal in their diet compared to those that had high proportions; six of these taxa were significantly reduced in the low bearded seal diet, and one was significantly increased. The largest difference in bacterial abundance was a 9.13 log fold decrease of DENOVO121 (Family; Peptostreptococcaceae, Genus; *Clostridium XI*) in the diet of individuals with a low proportion of bearded seal. A 5.47 log fold decrease in the bacterial abundance of DENOVO78 (Family; Rhodobacteraceae, Genus; *Paracoccus*) was also seen in the diet of individuals with a low proportion of bearded seal. A similar decrease in bacterial abundance was seen in DENOVO14 (Family; Peptostreptococcaceae, Genus; *Romboutsia*; 5.03 log fold decrease) with a low proportion of bearded seal. The only OTU that significantly increased in abundance with a low proportion of bearded seal was DENOVO29 (Family; Veillonellaceae, Genus; *Dialister*; 3.06 log fold increase). Only one OTU significantly differed with proportion of ringed seal in the diet, where a 3.40 log fold change in the abundance of DENOVO5 (Family; Peptostreptococcaceae, Genus; *Clostridium XI*) was detected in individuals with low proportions of ringed seal in the diet.

**Table 1:** OTUs that significantly differed in abundance within the gut microbiota of polar bears depending on diet type consumed (as shown in Fig. 3).

OTU	Phylum	Family	Genus
DENOVO5	Firmicutes	Peptostreptococcaceae	*Clostridium XI*
DENOVO14	Firmicutes	Peptostreptococcaceae	*Romboutsia*
DENOVO16	Bacteroidetes	Flavobacteriaceae	*NA*
DENOVO29	Firmicutes	Veillonellaceae	*Dialister*
DENOVO54	Actinobacteria	Actinomycetaceae	*Flaviflexus*
DENOVO59	Firmicutes	Erysipelotrichaceae	*Clostridium XVIII*
DENOVO78	Proteobacteria	Rhodobacteraceae	*Paracoccus*
DENOVO121	Firmicutes	Peptostreptococcaceae	*Clostridium XI*

Taxonomic information for each OTU is listed to genus level.

## Discussion

The gut microbiota may modulate the toxicity of environmental contaminants within a host (see Dempsey et al.^3^) and, conversely, contaminants may also impact gut bacteria communities^9,47^. Here we have demonstrated that diet-driven mercury concentrations are associated with differences in the diversity and composition of polar bear (*Ursus maritimus*) gut bacterial communities. We found bacterial alpha diversity to be low when the proportion of dietary seal consumption and THg levels were high. This result was detected independent of host ecological characteristics; bear age class, sex or body condition (despite previous research indicating that mercury concentrations vary with sex and age class in polar bears^30^). Additionally, we found that, independently, THg levels and the seal species consumed [ringed seal (*Pusa hispida*) or bearded seal (*Erignathus barbatus*)], were associated with significant differences in the abundance of a number of bacterial taxa.

Our finding that bacterial diversity had a negative relationship with THg levels mirrors previous work in the isopod *Porcellio scaber*, whereby individuals from mercury contaminated, compared to uncontaminated, natural environments demonstrated a lower bacterial species richness in the gut^9^. However, our findings contrast with reports from laboratory mice and pregnant women, showing no significant difference in bacterial diversity was associated with heavy metal exposure (i.e. cadmium, lead or mercury^4,48,49^). Our results also contrast findings in zebrafish (*Danio rerio*) experimentally exposed to lead, in which bacterial diversity instead significantly increased^47^. Therefore, in this study, our findings more closely mirror what is detected in natural conditions compared to a laboratory setting, perhaps indicating that experimentally derived results do not accurately reflect how contaminants impact the host microbiota in the wild. As previous studies have highlighted (see Pascoe et al.^50^), this study again demonstrates the importance of obtaining microbiota data from free-roaming wild species in addition to captive or laboratory animals, or humans. It is possible that the low bacterial diversity we detected with higher mercury levels may demonstrate that a number of commensal gut bacteria within polar bears are not mercury tolerant or, alternatively, that the polar bear gut microbiota responds negatively to contaminant-induced stress.

In addition to finding a low bacterial diversity associated with high THg levels, we also detected variation in the composition and abundance of bacterial taxa associated with THg levels. Gilmour et al.^51^ demonstrated that a number of bacterial species are capable of Hg methylation (a process which has been linked to the specific gene cluster; hgcAB), including bacterial species belonging to fermentative Firmicutes. Similarly, a number of studies have demonstrated the ability of gut bacteria to transform Hg within the intestine, something which has been demonstrated in aquatic organisms, terrestrial invertebrates, and mammals (see Li et al.^52^). It would appear that the genetic determinants (namely the hgcAB genes) of specific microbial taxa may confer resistance to mercury and, as such, hgcAB homologues may be important predictors for Hg methylation potential^51,52^. In our study, we found higher median proportions of Firmicutes and Proteobacteria, but lower median proportions of Bacteroidetes and Actinobacteria, in individuals above the safe threshold for mercury. This finding is similar to previous studies in laboratory mice that have been exposed to lead^49^, arsenic and iron^53^, where the abundance of Firmicutes were higher, while Bacteroidetes abundance was reduced. However, Wu et al.^49^ found no differences in the abundance of Actinobacteria and Proteobacteria with heavy metal exposure, unlike what we found in this study.

The ratio of Bacteroidetes:Firmicutes is considered an important indicator of mammalian health, including body mass, with the ratio of Bacteroides:Firmicutes increasing as fat mass decreases^54,55^. As such, changes to the Bacteroides:Firmicutes ratio may influence the capacity of a host to harvest energy from the diet. However, it is important to note that some studies have been unable to confirm a correlation between Bacteroides:Firmicutes ratio and host health^39,56^. At order level, one of the largest differences in bacterial abundance between bears with high versus low mercury levels was seen in Lactobacillales (which was higher in individuals above the safe threshold for mercury). Some strains of lactobacilli are able to methylate mercury, perhaps explaining its higher abundance in polar bears with high mercury levels^57^, and studies in mice have demonstrated elevated abundances of *Lactobacillus* sp. in individuals exposed to a combination of arsenic and iron^53^. We also found the abundance of Bacillales was greater in polar bears with high levels of mercury contamination. Members of the order Bacillales are able to generate highly resistant dormant spores under conditions of nutrient depletion and can survive in the absence of nutrients, amongst other harsh environmental conditions, for long periods of time^58^. It has also been demonstrated that spores from members of the order Bacillales are resistant to a number of toxic chemicals^59^, again explaining their higher abundance in polar bears with high mercury levels. Further, members of Lactobacillales and Bacillales are considered efficient in preventing (or restoring gut health following) intestinal disorders, such as colitis and Irritable Bowel Syndrome (IBS), at least in humans (see Ilinskaya et al.^60^). We therefore speculate whether the high abundance of Lactobacillales and Bacillales found within polar bears with high mercury levels could potentially reflect attempts to restore gut health from the stress of mercury contamination. However, further research would need to be conducted in order to draw a firmer conclusion on this. Conversely, we found the abundance of Bacteroidales was lower in polar bears demonstrating high THg levels. Members of the order Bacteroidales respond negatively to mercury exposures^61^, which is a concern considering that members of Bacteroidales have been shown to stimulate intraepithelial lymphocytes within the epithelial barrier, which promotes barrier integrity and protects the host from microbial invasion^62^. Interestingly, we also found a lower abundance of Selenomonadales in polar bears that had high THg levels, despite the fact that members of Selenomonadales have been shown to be mercury resistant^63^. Such mercury-dependent differences in microbiota composition are concerning, especially considering how the elevated mercury levels are acquired predominantly through consumption the polar bear’s main prey choice, seal^15^.

As the largest, most carnivorous ursid species, polar bears have evolved to hunt lipid-rich marine mammals to satisfy the high energy requirements associated with living in the challenging Arctic climate^64^. However, the decreased bacterial diversity we found linked with greater dietary intake of seal and associated increased hair mercury concentrations may indicate that the consumption of typical polar bear prey items is detrimental for polar bear gut health and resilience. A high bacterial diversity within the gut is typically associated with increased resilience and enhanced host health, and a low bacterial diversity (as we see in high mercury individuals within this study) the converse^65,66^. Further, we found that the consumption of different seal species was associated with differences in gut microbiota composition and abundance. The largest difference in bacterial abundance was seen in the genus *Clostridium XI* (Family; Peptostreptococcaceae) which was significantly higher in the diet of individuals with a high proportion of bearded seal. *Clostridium XI* is associated with high-fat diets and assimilating weight gain^67,68^, which perhaps explains its diminished proportions in individuals demonstrating low dietary intake of bearded seal (i.e. a dietary item rich in blubber). Similarly, we detected a significantly increased abundance of the genus *Paracoccus* (Family; Rhodobacteraceae) in polar bears that consume a high dietary intake of bearded seal. The family Rhodobacteraceae is among the most widely distributed bacterial lineages in marine habitats^69,70^. The only OTU that significantly increased in abundance with a low proportion of bearded seal in polar bear diet was DENOVO29 (Family; Veillonellaceae, Genus; *Dialister*). The abundance of bacteria belonging to the family Veillonellaceae, including the genus *Dialister* is positively correlated with dietary fatty acid intake in laboratory mice, and are thought to play an important role in energy metabolism^71^. Only one OTU significantly differed with proportion of ringed seal in the diet, DENOVO5 (Family; Peptostreptococcaceae, Genus; *Clostridium XI*), which was detected in a higher abundance in individuals with low proportions of ringed seal in the diet.

Diet, in itself, is considered a central driver in changes to the gut microbiota; gut microbiota analysis of humans and 59 other mammalian species indicates that host diet has a strong influence on bacterial diversity and composition^21^. Our findings indicate that diet influences gut microbiota diversity and composition, and reflects empirical observations in other ursids. In free-roaming brown bears (*U. arctos*), gut microbiota composition changes seasonally, which is thought to reflect extreme seasonal shifts in dietary intake^22^. Similarly, in Andean bears (*Tremarctos ornatus*), gut microbiota richness differs between captive and wild animals, which is thought to reflect differences in the availability and diversity of food resources^23^.

Overall, within this study we have shown that diet driven mercury contamination may have more intricate effects on host health than previously shown under laboratory conditions, with high seal consumption and associated high mercury concentrations associated with low diversity and particular composition of polar bear gut microbiota. It is important to note that within this study we only investigated the effect of one heavy metal (THg) on the polar bear gut microbiota. A combination of different heavy metals and other pollutants may have varying or even accumulative effects on gut diversity and composition compared to examining THg alone. Given that polar bears are apex predators and are vulnerable to multiple environmental stressors, they are considered a useful indicator species for the health of Arctic ecosystems. A deeper understanding of factors that influence polar bear intestinal health will feed into the development of future conservation strategies aiming to improve the persistence and resilience of this specialized high trophic feeding carnivore. We highlight the need to understand the interaction of multiple stressors, namely climate change, diet and contaminants, and their influence on the gut microbiota and health of Arctic wildlife.

## Supplementary Information

Supplementary Information.

## Data Availability

PRJNA542176.
