# Supplementary material for: Diet-driven mercury contamination is associated with polar bear gut microbiota
Source: Sci Rep. 2021 Dec 3;11:23372. doi: 10.1038/s41598-021-02657-6 (PMC8642428; doi:10.1038/s41598-021-02657-6)
Supplement: Supplementary file 1 — Supplementary Information. [file 41598_2021_2657_MOESM1_ESM.pdf]

Diet-driven mercury contamination is associated with polar bear gut microbiota

Sophie E. Watson, Melissa A. McKinney, Massimo Pindo, Matthew J. Bull, Todd C. Atwood, Heidi C. Hauffe & Sarah E. Perkins

Supplementary Table 1. Metadata associated with 91 southern Beaufort Sea polar bears for which faecal samples were collected for gut microbiota analysis. Individuals are separated by those for which i) mercury data was collected, ii) diet data was collected, and iii) both mercury and diet data was collected. Polar bear ID refers to the code assigned to each individual polar bear by the USGS.

Individuals with mercury data only							
Sample	Polar bear ID	Year	Age Class	Sex	Mercury data available	Mercury data source	Diet data available
1	20413	2008	Adult	Female	Yes	McKinney et al. 2017a	No
2	20845	2009	Adult	Female	Yes	McKinney et al. 2017a	No
3	20573	2009	Adult	Female	Yes	McKinney et al. 2017a	No
4	20457	2009	Adult	Male	Yes	McKinney et al. 2017a	No
5	20932	2010	Adult	Male	Yes	McKinney et al. 2017a	No
6	21024	2013	Adult	Female	Yes	Current study	No
7	20734	2013	Adult	Female	Yes	Current study	No
8	20521	2013	Adult	Female	Yes	Current study	No
9	21351	2013	Adult	Female	Yes	Current study	No
10	21358	2013	Adult	Female	Yes	Current study	No
11	20807	2013	Adult	Female	Yes	Current study	No
12	32227	2013	Adult	Female	Yes	Current study	No
13	20525	2013	Adult	Female	Yes	Current study	No
14	21361	2013	Adult	Female	Yes	Current study	No
15	32821	2013	Adult	Female	Yes	Current study	No
16	20961	2013	Adult	Female	Yes	McKinney et al. 2017a	No
17	21336	2013	Adult	Female	Yes	McKinney et al. 2017a	No
18	21339	2013	Adult	Female	Yes	McKinney et al. 2017a	No
19	20201	2013	Adult	Female	Yes	McKinney et al. 2017a	No
20	21333	2013	Adult	Female	Yes	McKinney et al. 2017a	No
21	20300	2013	Adult	Male	Yes	Current study	No
22	20206	2013	Adult	Male	Yes	Current study	No
23	21222	2013	Adult	Male	Yes	Current study	No
24	20886	2013	Adult	Male	Yes	Current study	No
25	20642	2013	Adult	Male	Yes	Current study	No
26	20580	2013	Adult	Male	Yes	Current study	No
27	21256	2013	Adult	Male	Yes	Current study	No
28	20556	2013	Adult	Male	Yes	Current study	No
29	21337	2013	Adult	Male	Yes	McKinney et al. 2017a	No
30	20710	2013	Adult	Male	Yes	McKinney et al. 2017a	No
31	21344	2013	Adult	Male	Yes	McKinney et al. 2017a	No
32	21328	2013	Adult	Male	Yes	McKinney et al. 2017a	No
33	21330	2013	Adult	Male	Yes	McKinney et al. 2017a	No
34	21329	2013	Adult	Male	Yes	McKinney et al. 2017a	No
35	21347	2013	Subadult	Female	Yes	Current study	No
36	21214	2013	Subadult	Female	Yes	Current study	No
37	21349	2013	Subadult	Female	Yes	Current study	No
38	21359	2013	Subadult	Male	Yes	Current study	No
39	21360	2013	Subadult	Male	Yes	Current study	No
40	21341	2013	Subadult	Male	Yes	Current study	No
41	21047	2013	Subadult	Male	Yes	McKinney et al. 2017a	No

Individuals with diet data only							
Sample	Polar bear ID	Year	Age Class	Sex	Mercury data available	Mercury data source	Diet data available
42	20981	2008	Adult	Female	No	NA	Yes
43	20966	2008	Adult	Female	No	NA	Yes
44	20974	2008	Adult	Female	No	NA	Yes
45	20741	2008	Adult	Female	No	NA	Yes
46	20751	2008	Adult	Male	No	NA	Yes
47	32255	2008	Subadult	Female	No	NA	Yes
48	20975	2008	Subadult	Male	No	NA	Yes
49	20982	2008	Subadult	Male	No	NA	Yes
50	20534	2009	Adult	Female	No	NA	Yes
51	20493	2009	Adult	Female	No	NA	Yes
52	20473	2009	Adult	Male	No	NA	Yes
53	20987	2009	Adult	Male	No	NA	Yes
54	20497	2009	Adult	Male	No	NA	Yes
55	20988	2009	Adult	Male	No	NA	Yes
56	20990	2009	Adult	Male	No	NA	Yes
57	20968	2009	Subadult	Male	No	NA	Yes
58	20986	2009	Subadult	Male	No	NA	Yes
59	21032	2009	Subadult	Male	No	NA	Yes
60	21015	2010	Adult	Female	No	NA	Yes
61	20859	2010	Adult	Female	No	NA	Yes
62	20414	2010	Adult	Female	No	NA	Yes
63	21221	2010	Adult	Female	No	NA	Yes
64	21045	2010	Adult	Female	No	NA	Yes
65	32366	2010	Adult	Female	No	NA	Yes
66	20125	2010	Adult	Male	No	NA	Yes
67	20424	2010	Adult	Male	No	NA	Yes
68	20606	2010	Adult	Male	No	NA	Yes
69	20475	2010	Adult	Male	No	NA	Yes
Individuals with both mercury and diet data							
Sample	Polar bear ID	Year	Age Class	Sex	Mercury data available	Mercury data source	Diet data available
70	20586	2008	Adult	Female	Yes	McKinney et al. 2017a	Yes
71	32606	2008	Adult	Female	Yes	McKinney et al. 2017a	Yes
72	6700	2008	Adult	Female	Yes	McKinney et al. 2017a	Yes
73	20446	2008	Adult	Female	Yes	McKinney et al. 2017a	Yes
74	20985	2008	Subadult	Female	Yes	McKinney et al. 2017a	Yes
75	32608	2008	Subadult	Male	Yes	McKinney et al. 2017a	Yes
76	6810	2009	Adult	Female	Yes	McKinney et al. 2017a	Yes
77	20764	2009	Adult	Female	Yes	McKinney et al. 2017a	Yes
78	20991	2009	Adult	Male	Yes	McKinney et al. 2017a	Yes
79	20733	2009	Adult	Male	Yes	McKinney et al. 2017a	Yes
80	20571	2009	Adult	Male	Yes	McKinney et al. 2017a	Yes
81	20977	2009	Subadult	Female	Yes	McKinney et al. 2017a	Yes
82	20485	2010	Adult	Female	Yes	McKinney et al. 2017a	Yes
83	21157	2010	Adult	Female	Yes	McKinney et al. 2017a	Yes
84	21219	2010	Adult	Female	Yes	McKinney et al. 2017a	Yes
85	21000	2010	Adult	Female	Yes	McKinney et al. 2017a	Yes
86	20195	2010	Adult	Male	Yes	McKinney et al. 2017a	Yes
87	32376	2010	Adult	Male	Yes	McKinney et al. 2017a	Yes
88	20449	2010	Adult	Male	Yes	McKinney et al. 2017a	Yes
89	21127	2010	Adult	Male	Yes	McKinney et al. 2017a	Yes
90	21022	2010	Subadult	Male	Yes	McKinney et al. 2017a	Yes
91	20972	2010	Subadult	Male	Yes	McKinney et al. 2017a	Yes

Supplementary Table 2. Results of the GLM showing that alpha diversity indices of polar bear faecal microbiota were not significantly different by sex, age class or body condition, but only according to an interaction between total hair mercury (THg) and total seal consumption. Asterisks indicate significant terms.

	Shannon				Inverse Simpson			
	Deviance	AIC	F-value	P-value	Deviance	AIC	F-value	P-value
**Body mass index**	0.40	30.03	1.04	0.32	0.28	108.77	0.03	0.86
**Age class**	0.43	29.33	0.84	0.37	0.29	111.64	0.31	0.58
**Sex**	0.48	29.61	2.04	0.17	0.36	113.65	4.02	0.06
**Mercury : Total seal consumption**	0.76	38.69	8.00	0.013*	0.66	123.37	6.04	0.027*

Supplementary Table 3. Log fold change in OTU abundance within the gut microbiota of polar bears demonstrating mercury levels above, compared to below, NOEL threshold for humans

OTU	Phylum	Family	Genus	Log fold change in abundance
**DENOVO41**	Proteobacteria	Moraxellaceae	*Acinetobacter*	-10.74
**DENOVO24**	Proteobacteria	Alcaligenaceae	*Oligella*	4.21
**DENOVO46**	Firmicutes	Veillonellaceae	*Megamonas*	-5.45
**DENOVO15**	Firmicutes	Veillonellaceae	*Megamonas*	-3.01
**DENOVO47**	Firmicutes	Staphylococcaceae	*Nosocomiicoccus*	3.62
**DENOVO20**	Firmicutes	Erysipelotrichaceae	*Turicibacter*	-4.68
**DENOVO32**	Firmicutes	Clostridiaceae 1	*Clostridium sensu stricto*	-2.78
**DENOVO27**	Firmicutes	Lachnospiraceae	*Clostridium XlV*	-1.82
**DENOVO55**	Firmicutes	Ruminococcaceae	*NA*	-4.60
**DENOVO14**	Firmicutes	Peptostreptococcaceae	*Romboutsia*	-2.69
**DENOVO121**	Firmicutes	Peptostreptococcaceae	*Clostridium XI*	-4.83
**DENOVO21**	Firmicutes	Peptostreptococcaceae	*Peptostreptococcus*	-3.04
**DENOVO9**	Firmicutes	Clostridiales_Incertae Sedis XI	*Anaerococcus*	-3.31
**DENOVO38**	Firmicutes	Clostridiales_Incertae Sedis XI	*Murdochiella*	-9.29
**DENOVO23**	Firmicutes	Clostridiales_Incertae Sedis XI	*Murdochiella*	-4.00
**DENOVO115**	Bacteroidetes	Bacteroidaceae	*Bacteroides*	-4.27
**DENOVO82**	Bacteroidetes	Bacteroidaceae	*Bacteroides*	-2.81
**DENOVO17**	Bacteroidetes	Bacteroidaceae	*Bacteroides*	-2.93
